# Proteome Analysis Identifies the Dpr Protein of *Streptococcus mutans* as an Important Factor in the Presence of Early Streptococcal Colonizers of Tooth Surfaces

**DOI:** 10.1371/journal.pone.0121176

**Published:** 2015-03-27

**Authors:** Akihiro Yoshida, Mamiko Niki, Yuji Yamamoto, Ai Yasunaga, Toshihiro Ansai

**Affiliations:** 1 Department of Oral Microbiology, Matsumoto Dental University, Shiojiri, Japan; 2 Division of Community Oral Health Science, Department of Oral Health Promotion, Kyushu Dental University, Kitakyushu, Japan; 3 Department of Bacteriology, Osaka City University Graduate School of Medicine, Osaka, Japan; 4 Department of Animal Science, School of Veterinary Medicine, Kitasato University, Towada, Japan; University of Kansas Medical Center, UNITED STATES

## Abstract

Oral streptococci are primary colonizers of tooth surfaces and *Streptococcus mutans* is the principal causative agent of dental caries in humans. A number of proteins are involved in the formation of monospecies biofilms by *S*. *mutans*. This study analyzed the protein expression profiles of *S*. *mutans* biofilms formed in the presence or absence of *S*. *gordonii*, a pioneer colonizer of the tooth surface, by two-dimensional gel electrophoresis (2-DE). After identifying *S*. *mutans* proteins by Mass spectrometric analysis, their expression in the presence of *S*. *gordonii* was analyzed. *S*. *mutans* was inoculated with or without *S*. *gordonii* DL1. The two species were compartmentalized using 0.2-μl Anopore membranes. The biofilms on polystyrene plates were harvested, and the solubilized proteins were separated by 2-DE. When *S*. *mutans* biofilms were formed in the presence of *S*. *gordonii*, the peroxide resistance protein Dpr of the former showed 4.3-fold increased expression compared to biofilms that developed in the absence of the pioneer colonizer. In addition, we performed a competition assay using *S*. *mutans* antioxidant protein mutants together with *S*. *gordonii* and other initial colonizers. Growth of the *dpr*-knockout *S*. *mutans* mutant was significantly inhibited by *S*. *gordonii*, as well as by *S*. *sanguinis*. Furthermore, a cell viability assay revealed that the viability of the *dpr*-defective mutant was significantly attenuated compared to the wild-type strain when co-cultured with *S*. *gordonii*. Therefore, these results suggest that Dpr might be one of the essential proteins for *S*. *mutans* survival on teeth in the presence of early colonizing oral streptococci.

## Introduction

The development of dental caries is a complex process which is dependent on a presence of microbial biofilm known as dental plaque [1]. Of the oral bacteria which compose the oral biofilm, *Streptococcus mutans* has been considered as the bacterial species most closely associated with initiation of human dental caries [2]. Oral bacteria form a biofilm on the tooth surface that accumulates through the sequential and ordered colonization of more than 500 different species of bacteria [2]. Bacteria comprise early, middle, or late colonizers that undergo successive attachment of saliva-suspended species to previously attached bacteria and form multispecies communities [3, 4]. Initial colonizers bind to host-derived receptors on the salivary pellicle of the tooth enamel. Of these bacteria, the oral commensals *S*. *gordonii* and *S*. *sanguinis* are representative pioneer colonizers of the pellicle [5, 6]. In addition, *S*. *sanguinis* and *S*. *gordonii* use oxygen and hydrogen peroxide (H_2_O_2_) to compete against *S*. *mutans* [7]. Moreover, the proteases of *S*. *gordonii* interfere with subsequent colonization by *S*. *mutans* [8] and bacteriocin production by *S*. *mutans* is also inhibited by *S*. *gordonii* [9]. Clinical studies have also indicated that *S*. *sanguinis* and *S*. *gordonii* can antagonize *S*. *mutans* colonization when present in oral biofilms in high numbers [10].

*S*. *gordonii* is a key pioneer colonizer and can also affect the initial attachment of *S*. *mutans* to the tooth surface. Studies have reported that interspecies interactions are mediated through chemicals (*e*.*g*., bacteriocin, H_2_O_2_, and protease) produced by *S*. *gordonii* [8, 11–14]. However, it is not yet fully understood how interspecies interactions with early streptococcal colonizers affect *S*. *mutans* colonization. Indeed, *S*. *mutans* still exists in the oral biofilms on tooth surfaces even when exposed to potential inhibitors produced by *S*. *gordonii*. The objectives of this study were to determine the resistance mechanisms of *S*. *mutans* relative to competition with *S*. *gordonii* in the initial stages of biofilm formation.

## Materials and Methods

### Bacterial strains and growth conditions

The *S*. *mutans* UA159, *S*. *mutans* GS5, *S*. *gordonii* DL1 (Challis), and their derivative strains used in this study are listed in Table 1. All strains were maintained aerobically (5% CO_2_) or in an anaerobic chamber (90% N_2_, 5% CO_2_, and 5% H_2_) at 37°C in brain heart infusion (BHI) medium (Becton Dickinson, Sparks, MD), Todd-Hewitt broth (THB, Becton Dickinson), or on THB agar plates. For biofilm formation, chemically defined medium (CDM) was used [15]. The CDM contained 2.0 g l^−1^of L-glutamic acid, 0.2 g l^-1^ of L-cysteine, 0.9 g l^-1^ of L-leucine, 1.0 g l^-1^ of NH_4_Cl, 2.5 g l^-1^ of K_2_HPO_4_, 2.5 g l^-1^ of KH_2_PO_4_, 4.0 g l^-1^ of NaHCO_3_, 1.2 g l^-1^ of MgSO_4_·7H_2_O, 0.02 g l^-1^ of MnCl_2_·4H_2_O, 0.02 g l^-1^ of FeSO_4_·7H_2_O, 0.6 g l^-1^ of sodium pyruvate, 1.0 mg l^-1^ of riboflavin, 0.5 mg l^-1^ of thiamine HCl, 0.1 mg l^-1^ of D-biotin, 1.0 mg l^-1^ of nicotinic acid, 0.1 mg l^-1^ of *p*-aminobenzoic acid, 0.5 mg l^-1^ of Ca-pantothenate, 1.0 mg l^-1^ of pyridoxal HCl, and 0.1 mg l^-1^ of folic acid, adjusted to pH 7.0 with H_3_PO_4_. For antibiotic selection, cultures were supplemented with the following antibiotics: 250 μg ml^-1^ spectinomycin for *S*. *mutans*, 10 μg ml^-1^ erythromycin for *S*. *gordonii*, and 100 μg ml^-1^ ampicillin for *Escherichia coli*.

**Table 1:** The strains and plasmids used in this study.

Strain or plasmid	Relevant characteristics	Source or reference
Strains (original name)		
*Streptococcus mutans*		
UA159	WT laboratory strain, Em^s^ ^*a*^	KDU^*f*^
UA159 Δ*dpr*	UA159, *dpr*::Em^r^ ^*b*^	This study
GS5	WT laboratory strain, Em^s^	KDU
GS5 Δ*dpr* (DES)	GS5, *dpr*::Spc^r^ ^*c*^	[20]
GS5 Δ*dpr*+*dpr*	*dpr* complementation in GS5 Δ*dpr*, Spc^r^, Em^r^, Km^r^ ^*d*^ (GS5 Δ*dpr* harboring pAY1301)	This study
GS5 Δ*sod* (KD251)	GS5, *sod*::Em^r^	[20]
GS5 Δ*ahpC* (BEE)	GS5, *ahpC*::Em^r^::*nox-1*	[20]
GS5 Δ*dpr*+ Δ*sod* (KD251-DES)	GS5, *dpr*::Spc^r^, *sod*::Em^r^	[20]
GS5 Δ*dpr*+ Δ*ahpC* (BEE-DES)	GS5, *dpr*::Spc^r^, *ahpC*::Em^r^::*nox-1*	[20]
*Escherichia coli* DH5α	Cloning host	[39]
*Streptococcus gordonii*		
DL1/Challis	WT laboratory strain, Em^s^	KDU
DL1 Δ*spxB*	DL1, *spxB*::Em^r^	This study
*Streptococcus oralis* ATCC 10557	Oral commensal	RIKEN^*g*^
*Streptococcus mitis* ATCC 49456	Oral commensal	RIKEN
*Streptococcus salivarius* HHT	Oral commensal	RIKEN
*Streptococcus sanguinis* ATCC 10556	Oral commensal	RIKEN
*Actinomyces naeslundii* JCM8350	Oral commensal	RIKEN
Plasmids		
pDL276	*Streptococcus-E*. *coli* shuttle plasmid, Km^r^	[21]
pResEmMCS10	*Streptococcus* integration plasmid, Em^r^, Amp^r^ ^*e*^	[40]
pAY1201	pResEmMCS10 harboring the upstream and downstream regions of *S*. *mutans* UA159 *dpr*, Em^r^, Amp^r^	This study
pAY2201	pResEmMCS10 harboring the upstream and downstream region of *S*. *gordonii* DL1 *spxB*, Em^r^, Amp^r^	This study
pAY1301	pDL276 harboring *S*. *mutans* GS5 *dpr* and PCR-generated Em^r^ gene, Em^r^, Km^r^	This study

^*a*^ Em^s^, erythromycin-sensitive.

^*b*^ Em^r^, erythromycin-resistant.

^*c*^ Spc^r^, spectinomycin-resistant.

^*d*^ Km^r^, kanamycin-resistant.

^*e*^ Amp^r^, ampicillin-resistant

^*f*^. KDU, Culture collection of the Division of Community Oral Health Science, Kyushu Dental University, Kitakyushu, Japan.

^*g*^ RIKEN, Microbe Division/Japan Collection of Microorganisms, RIKEN BioResource Center, Wako, Japan.

### DNA manipulations

Routine molecular biology techniques were basically performed as previously described [16]. PCR products were purified using a QIAquick PCR purification kit (QIAGEN, Valencia, CA). Chromosomal DNA was isolated from the bacteria listed in Table 1 using a Puregene DNA isolation kit (Gentra Systems, Minneapolis, MN). Nucleotide sequence information for *S*. *mutans* and *S*. *gordonii* were obtained from the Oral Pathogen Sequence Database (Los Alamos National Laboratory, http://www.oralgen.lanl.gov/).

### Biofilm formation

*S*. *mutans* UA159 was inoculated with *S*. *gordonii* DL1 using a two-compartment system [17, 18] with a slight modification. Briefly, each well of a six-well polystyrene plate (Corning Inc., Corning, NY) was separated into two compartments using Nunc 25-mm Tissue Culture Inserts with 0.2-μl Anopore membranes (Nunc, Roskilde, Denmark). Each compartment contained CDM supplemented with 0.5% sucrose. *S*. *gordonii* DL1 was inoculated in the upper compartment, and *S*. *mutans* UA159 was inoculated in the lower layer. As controls, *S*. *mutans* was inoculated in both the upper and lower compartments. Each overnight culture was added to 3 ml CDM (culture:CDM = 1:30) and incubated at 37°C under anaerobic conditions for 24 h. The *S*. *mutans* biofilm in the lower compartment was then collected for analysis.

### *S*. *mutans* whole-cell lysates

Samples to be subjected to two-dimensional gel electrophoresis (2-DE) were prepared using both chemical and mechanical extraction to ensure high yield and optimum solubility of whole-cell proteins. Biofilm cells on a six-well polystyrene plate (Corning) were harvested with a cell scraper (Asahi Glass, Tokyo, Japan) and washed four times with distilled water. The bacterial biofilm was suspended with 1.0 ml distilled water and disrupted using a Mini-Bead Beater (Biospec Products, Bartlesville, OK) with a 2 ml tube containing 0.1-mm-diameter silica sphere beads (Lysing Matrix B; MP Biomedicals LLC, Solon, OH) at 4800 rpm for 30 s. After disruption, the samples were cooled on ice for 3 min. This procedure was repeated five times. The aliquots were transferred to 1.5 mL tubes and centrifuged at 15,000 rpm for 5 min. The protein concentrations of the supernatant were measured (Quick Start Bradford Dye Reagent 1×; Bio-Rad, Hercules, CA) and the supernatant was subjected to acetone precipitation. A total of 200 μg protein per 1.5 ml tube was precipitated with 1.0 ml acetone and incubated at −30°C for more than 10 min. The samples were centrifuged at 15,000 rpm for 5 min, and the acetone was removed. Sample preparation was also performed with a 2-D Clean-Up Kit (GE Healthcare Bio-Sciences AB, Uppsala, Sweden), according to the manufacturer’s instructions.

### 2-DE. (i) Isoelectric focusing

Rehydration of Immobiline DryStrips (pH 4–7, 7 cm for preparative gels and 18 cm for analysis gels; GE Healthcare) and isoelectric focusing (IEF; first dimension) separation of proteins were performed using an Ettan IPGphor 3 system (GE Healthcare). The protein samples (120 μg for 7 cm and 300 μg for 18 cm) in DeStreak Rehydration Solution (130 μl for 7 cm and 320 μl for 18 cm, GE Healthcare) with IPG Buffer (pH 4–7, final 0.5% [vol/vol]; GE Healthcare) were loaded onto the strips. The strips were rehydrated and run in an Ettan IPGphor 3 instrument with an adequate length of strip holders (Ettan IPGphor 3 fixed-length strip holders, GE Healthcare). Rehydration was performed overnight at room temperature. The IEF parameters for 7-cm strips were as follows: (i) 0.5 h at 300 V (step and hold), (ii) 0.5 h at 1000 V (gradient), (iii) 5000 V for 1.5 h (gradient), and (iv) 5000 V for 36 min (step and hold). The IEF parameters for 18-cm strips were as follows: (i) 1 h at 500 V (step and hold), (ii) 1 h at 1000 V (gradient), (iii) 8000 V for 3 h (gradient), and (iv) 8000 V for 2 h 40 min (step and hold). All steps were performed at 20°C. **(ii) SDS-PAGE**. After IEF, the strips were initially equilibrated for 10 min with 10 ml SDS Equilibration buffer (50 mM Tris-HCl [pH 6.8]), 6 M urea, 30% [vol/vol] glycerol, 1% [wt/vol] SDS) containing 100 mg of dithiothreitol (*threo*-1,4-dimercapto-2,3-butanediol; DTT). Next, the strips were equilibrated for 10 min with 10 mL SDS Equilibration buffer containing 250 mg of iodoacetamide and 0.002% [wt/vol] bromophenol blue. Separation in the second dimension was carried out by standard SDS-PAGE by laying strips on 12.5% polyacrylamide gels (9 cm long × 1 mm wide × 8 cm high for 7-cm strips, and 20 cm long × 1.5 mm wide × 20 cm high for 18-cm strips) [19] for electrophoresis. The gels were stained with Coomassie brilliant blue G-250 (0.04% [wt/vol] Coomassie brilliant blue G-250, 3.5% [wt/vol] perchloric acid).

Quantification of protein changes across triplicates of the two conditions analyzed were captured via image analysis using Progenesis/SameSpot image analysis software (Nonlinear Dynamics, Newcastle upon Tyne, UK), and the average data of each spot were compared between two conditions. The spots in which the Norm volume was more than 1.5-fold and the differences were significant (*P* < 0.05, ANOVA) were selected for comparison analysis. Definition of the Norm volume was as follows: Norm volume = (volume of each spot) / (volumes of all spots)] × 100.

### MS analysis

Spots from the 2-DE analyses were submitted to in-gel proteolysis and LC-MS/MS (APRO Science, Tokushima, Japan). The gel pieces were washed twice and the proteins were dehydrated in the gel with acetonitrile, rehydrated with 10% acetonitrile in 10 mM Tris-HCl (pH 8.0) containing trypsin, and incubated at 35°C for 20 h. Tryptic peptides were resolved by reverse-phase chromatography on 0.1- × 50-mm fused silica capillaries (L-column ODS; Chemicals Evaluation and Research Institute [CERI], Tokyo, Japan). The peptides were eluted with linear gradients of 2% to 95% acetonitrile with 0.1% formic acid in water at flow rates of 0.5 μl/min. Mass spectroscopy (MS) was performed with an ion-trap mass spectrometer (Q-Tof2; Waters, Milford, MA) in positive mode using repetitive full MS scanning followed by collision-induced dissociation of the three most dominant ions selected from the first MS scan. Spot analysis was performed by LC-MS/MS combined with a search of the NCBInr database with Mascot software (Matrix Science Inc., Boston, MA).

### Plasmid construction

The *S*. *mutans dpr* gene was identified in the database (Oral Pathogen Sequence Database), and the promoter information was obtained from a previous investigation [20]. For the complementation analysis of the *S*. *mutans dpr* strain, PCR products generated using the primers dprF-Sal (pDL276) and dprR-Bam (pDL276) were inserted into pDL276 at *Sal*I and *BamH*I sites (Table 1, 2) [21]. To generate an erythromycin resistance gene, an erythromycin cassette was produced by PCR using the primer pair AM1 and AM3 [22]. The erythromycin cassette was ligated into pDL276 with the *dpr* fragment at the SmaI site; the resultant plasmid was designated pAY1301. To delete the *S*. *mutans* UA159 *dpr* gene, the plasmid was prepared as follows: Two fragments, up- and downstream of the *dpr* gene, were generated by PCR with the primers dprUF-Sal/dprUR-Pst and dprDF-Sac/dprDR-Kpn, respectively (Table 2). These products were cleaved with *SalI/PstI* and *Sac*I/*Kpn*I, respectively, and ligated into pResEmMCS10, resulting in pAY1201 (Table 1). To delete the *S*. *gordonii* DL1 *spxB* gene, which encodes the pyruvate oxidase SpxB protein, a plasmid was prepared as follows: two fragments, up- and downstream from the *spxB* gene, were generated by PCR with the primers spxBUF-Sal/spxBUR-Pst and spxBDF-Sac/spxBDR-Kpn, respectively (Table 2). These products were cleaved with *Sal*I/*Pst*I and *Sac*I/*Kpn*I, respectively, and ligated into pResEmMCS10, resulting in pAY2201 (Table 1).

**Table 2:** Primers used in this study.

Primer	Sequence^*a*^ (5’→3’)	Gene targeted
dprUF-Sal	GGGGGGGTCGACGAGGATTTGTCTACGCTG	*S*. *mutans dpr*
dprUR-Pst	GGGGGGCTGCAGCCTGATTAAGTACAGCC	
dprDF-Sac	GGGGGGGAGCTCATGTTGCAGGCAGAGCTT	*S*. *mutans dpr*
dprDR-Kpn	GGGGGGGGTACCGTTCCTACCTCTTGGGTA	
dprF-Sal (pDL276)	GGGGGGGTCGACAATCAGTCCGCAGAGTAA	*S*. *mutans dpr*
dprR-Bam (pDL276)	GGGGGGGGATCCTTATAAACCGGGAGCTTG	
spxBUF-Sal	CCCCCCGTCGACGTGATTGGCTTGATTGCC	*S*. *gordonii spxB*
spxBUR-Pst^*b*^	CTGATGGGATACCGTAGA	
spxBDF-Sac	GGGGGGGAGCTCGCCTCTTCTTGGAAGAAG	*S*. *gordonii spxB*
spxBDR-Kpn	GGGGGGGGTACCACACGCTACCATCTTCTG	
AM1 (Sma)	CCCCCCGGGGAAGGAGTGATTACATGAAC	Erythromycin cassette
AM3 (Sma)	CCCCCCGGGAGCGACTCATAGAATTATTTC	
RT-qPCR		
dprF	GTTCACCAAGTCCATTGG	*S*. *mutans dpr*
dprR	AAGGTTGAAAACGGAGCG	
sodF	TGGAACAAATTCCAGCGG	*S*. *mutans sod*
sodR	GCAGCTGTAAAAGCTGCT	
ahpCF	CGTGTGTCCTACTGAGTT	*S*. *mutans ahpC*
ahpCR	TGAGAAGGATCCCCAATC	
gyrAF	ATTGTTGCTCGGGCTCTTCCAG	*S*. *mutans gryA*
gyrAR	ATGCGGCTTGTCAGGAGTAACC	

^*a*^Endonuclease restriction sites are underlined.

^*b*^Restriction sites were not included.

### Transformation of *S*. *mutans* and *S*. *gordonii*

The *S*. *mutans* UA159 Δ*dpr* strain was constructed by allelic exchange via insertion of an erythromycin resistance determinant into the gene. The plasmid pAY1201 (Table 1), used for disruption of the *dpr* gene, was prepared as previously described. *S*. *mutans* Δ*dpr* strains containing pAY1301 were obtained by electrotransformation (Table 1) [23]. Genetic transformation of *S*. *gordonii* with linearized pAY2201 and synthetic CSP was performed as previously described (Table 1) [23]. The amino acid sequence of synthetic *S*. *gordonii* competence stimulating peptide (CSP) is DVRSNKIRLWWENIFFNKK (SIGMA Life Science, Ishikari, Japan) [24].

### Western blot analysis

Proteins from various extracts were resuspended in 5× Laemmli sample buffer [19]. Protein samples were separated by SDS-PAGE and electrotransferred to a polyvinylidene difluoride membrane. The membrane was blocked with TBS with 1% nonfat milk, reacted with a rabbit anti-*S*. *mutans* Dpr antibody, and subsequently developed with goat anti-rabbit IgG antibody conjugated to alkaline phosphatase [25]. The anti-Dpr antibodies were prepared from a rabbit immunized with the *S*. *mutans* Dpr preparation [25]. For the quantification of specific protein expression, total protein measurement was performed [26, 27].

### Competition assays on agar plates

Competition assays were basically performed as previously described with some modifications [7, 28]. Briefly, 5 μl of an overnight culture of either species adjusted to an optical density at 595 nm (OD_595_) of 0.5 in BHI was spotted on THB agar plates as the early colonizer. After overnight inoculation, 5 μl samples of *S*. *mutans* strains (3.5 × 10^6^ CFU) were spotted adjacent to the early colonizer strains, or both strains were simultaneously inoculated beside each other. The distance between the centers of the spots was 8 mm. The plates were further incubated at 37°C in anaerobic or aerobic (with or without 5% CO_2_, respectively) chambers.

### H_2_O_2_ sensitivity assay on agar plates

Various concentrations of 5 μl H_2_O_2_ were spotted on the THB agar plate, and 5 μl *S*. *mutans* strains (3.5 × 10^6^ CFU) were spotted adjacent to the H_2_O_2_. The distance between the centers of the spots was 8 mm.

### Transcriptional analysis by qPCR

*S*. *mutans* biofilm was collected and served for transcriptional analysis. Total RNA was extracted from *S*. *mutans* cells using by Isogen (Nippon Gene, Co. Ltd., Tokyo, Japan) according to manufacturer’s protocol. Reverse transcriptase reactions were performed by using ReverTra Ace (MMLV Reverse Transcriptase RNaseH-; Toyobo Co., Ltd., Osaka, Japan). The primer pairs used are listed in Table 2. The DNA gyrase A subunit (*gyrA*) was stably expressed and used as the internal control. Data were analyzed for statistically significant differences from the *S*. *mutans* alone control.

### *S*. *mutans* viability assay

To examine the viability of *S*. *mutans* strains after co-inoculation with *S*. *gordonii*, we inoculated pure cultures of *S*. *gordonii* overnight. A total of 10 ml overnight pure culture (OD_595_ = 0.8) was centrifuged, the supernatant was removed, and 9 ml fresh BHI medium were added to the bacterial pellet and the pellet was resuspended. A total of 1 ml of cultured *S*. *mutans* strains (2 × 10^8^ CFU/ml) was inoculated into the pre-existing *S*. *gordonii* medium and cultured at 37°C under anaerobic conditions for 48 h for subsequent colony counting.

### Statistical analysis

Student’s *t*-test, two-way ANOVA, and Bonferroni’s test were used to determine statistical significance. A difference was deemed significant at *P* < 0.05.

## Results

### 2-DE

**Fig 1:**
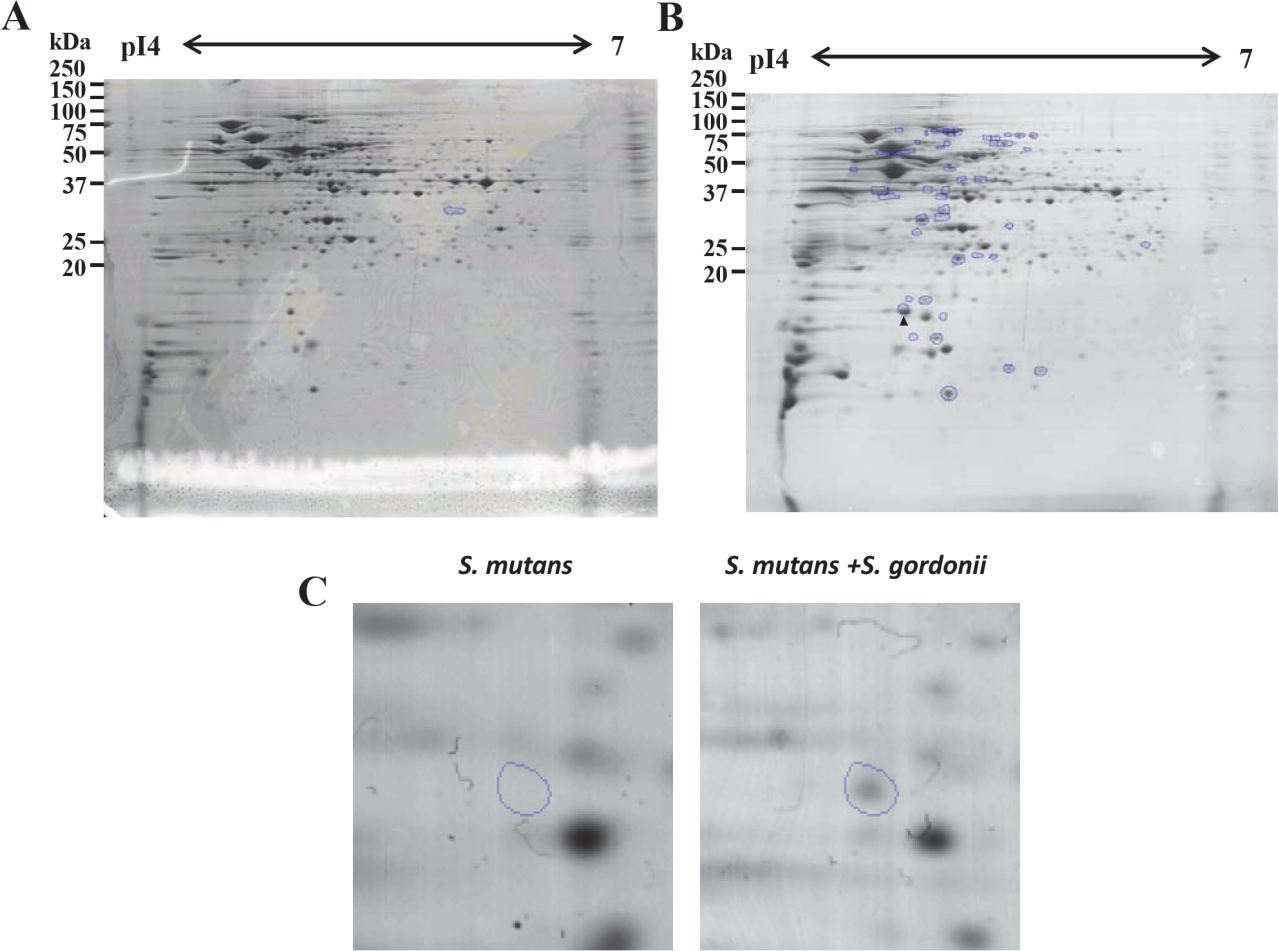
CBB-R250-stained 2-DE protein profiles of *S*. *mutans* UA159 biofilms on polystyrene plate co-cultured without (A) and with *S*. *gordonii* DL1 (B). The circled spot(s) are *S*. *mutans* UA159 monoculture biofilms proteins upregulated more than 1.5-fold compared to the *S*. *mutans* UA159 biofilms co-cultured with *S*. *gordonii* DL1 (A) or vice versa (B). The number of up-regulated protein was only one in *S*. *mutans* monoculture biofilm (A), whereas 46 protein spots were up-regulated in *S*. *mutans* co-cultured with *S*. *gordonii* (B). (C) The protein spot upregulated the most was No. 3633 (circled). The No. 3633 protein is indicated by arrowhead (B). Triplicate independent analysis for each sample were performed.

Comparison of CBB-R250-stained gels for the *S*. *mutans* UA159 biofilms with or without *S*. *gordonii* DL1 indicated that 46 protein spots of *S*. *mutans* were upregulated more than 1.5-fold when co-cultured with *S*. *gordonii* (*P* < 0.05), whereas only one protein spot was downregulated more than 1.5-fold in *S*. *mutans* cultured without *S*. *gordonii* (*P* < 0.05) (Fig. 1A, B). Additional protein spots were observed when co-cultured with *S*. *gordonii*. Of the 1209 detected protein spots for *S*. *mutans* biofilms, 1162 spots were not altered in the presence of *S*. *gordonii*. Of the 46 upregulated spots, the most upregulated spot was No. 3633 protein (4.3-fold) (Fig. 1C). LC-MS/MS indicated that spot No. 3633 was *S*. *mutans* Dpr, a peroxide resistance protein.

### Construction of *S*. *mutans dpr*-defective mutant and Western blotting analysis

To analyze the role of *S*. *mutans* Dpr when co-cultured with *S*. *gordonii*, the *dpr* gene (GenBank: SMU.540) of *S*. *mutans* UA159 was inactivated by allelic exchange mutagenesis. The resulting *S*. *mutans* UA159 Δ*dpr* grew very slowly under both aerobic and anaerobic conditions (S1 Fig.). Therefore, we used a *S*. *mutans* GS-5 *dpr* mutant to analyze Dpr [20]. For the complementation analysis, pAY1301, which contains the *dpr* gene, was transformed into the *S*. *mutans* GS5 Δ*dpr* strain by electroporation-mediated transformation. The expression of Dpr protein was confirmed by Western blotting. *S*. *mutans* GS5 and GS5 Δ*dpr*+*dpr* strains showed Dpr expression, but the GS5 Δ*dpr* strain did not (S2 Fig.).

### Competition between *S*. *mutans* and initial colonizers

**Fig 2:**
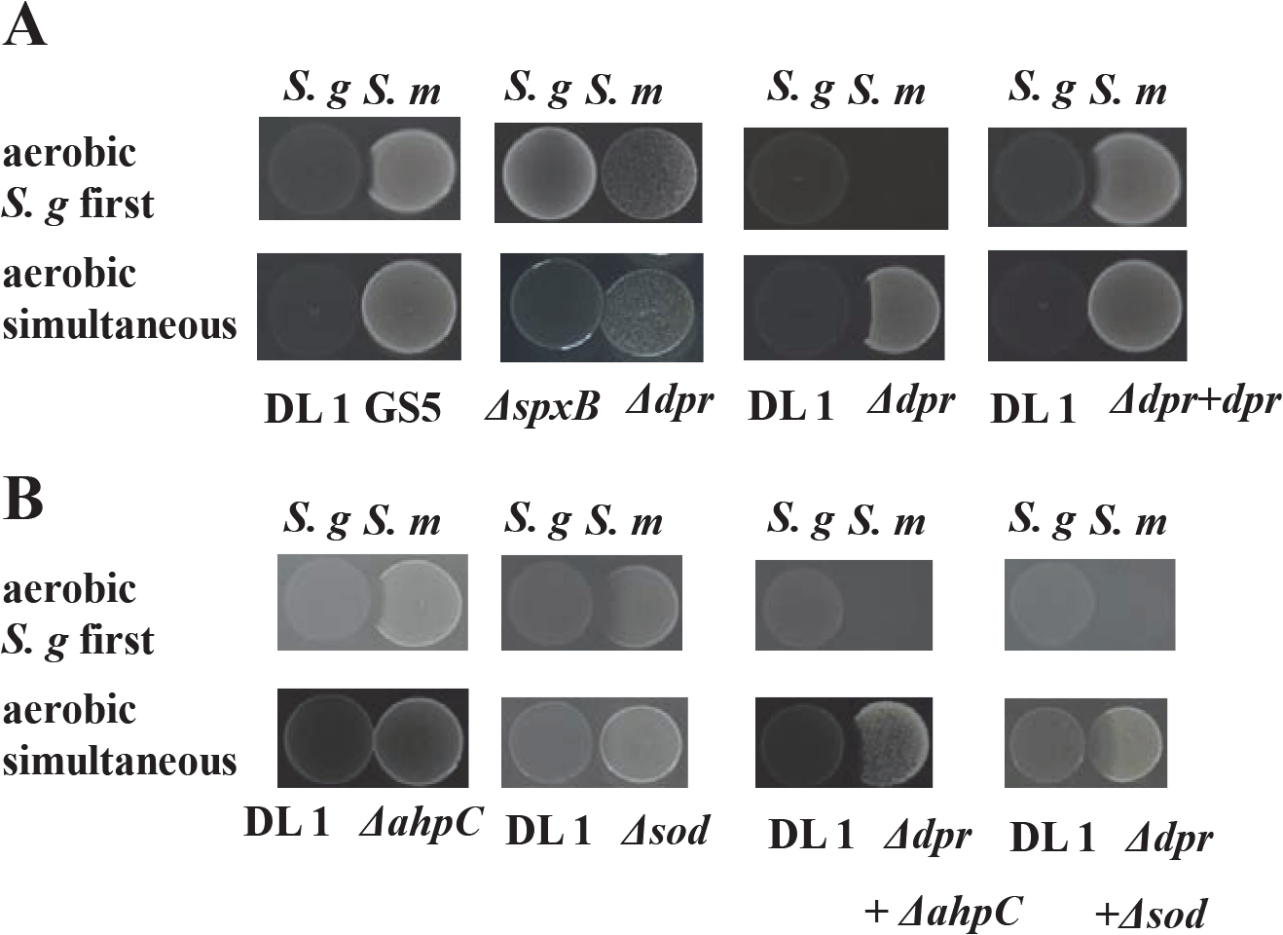
Inhibition of the growth of *S*. *mutans* strains by *S*. *gordonii*. (A) Inhibition of the growth of *S*. *mutans dpr*-deficient strains by *S*. *gordonii* DL1 and *spxB*-deficient strain. *S*. *gordonii* strains were inoculated first and grown for 24 h at 37°C in an aerobic atmosphere. Then, *S*. *mutans* strains were inoculated next to these colonizers, and the plates were incubated for 24 h (upper lane). *S*. *gordonii* and *S*. *mutans* were inoculated simultaneously on the plate and incubated for 24 h at 37°C under aerobic conditions (lower lane). (B) Inhibition of the growth of *S*. *mutans sod*-, *ahpC*-, *dpr*- *ahpC*, and *sod* double mutants by *S*. *gordonii* DL1. The culture conditions were the same as in (A).

Competition between *S*. *mutans* and initial colonizers was analyzed using two assays described by Kreth *et al*. [7]: (i) initial colonizer strains were inoculated and allowed to grow for 24 h before *S*. *mutans* strains were inoculated nearby, and (ii) both species were inoculated simultaneously. As shown in Fig. 2A, *S*. *gordonii* inhibited the growth of the *S*. *mutans dpr*-defective mutant in both conditions. No growth inhibition was observed in any *S*. *mutans* strains in the presence of the *S*. *gordonii spxB*-defective mutant in all conditions. In addition, the mutants encoding inactivated alkyl hydroperoxide reductase (AhpC) and superoxide dismutase (SOD) were analyzed (Fig. 2B). The growth of these mutants was not affected by *S*. *gordonii*, whereas double mutants (Δ*dpr* plus Δ*ahpC* and Δ*dpr* plus Δ*sod*) were inhibited more. The growth of *dpr*- *ahpC* and *sod* double mutants were more inhibited by *S*. *gordonii* compared to Δ*ahpC* and Δ*sod* strains, while slight or no inhibition was observed in Δ*ahpC* and Δ*sod* strains (Fig. 2B). The strains defective in Δ*ahpC*, Δ*sod*, Δ*dpr* plus Δ*ahpC*, and Δ*dpr* plus Δ*sod* were not inhibited by *S*. *gordonii spxB*-defective mutant in all conditions (S3 Fig.).

**Fig 3:**
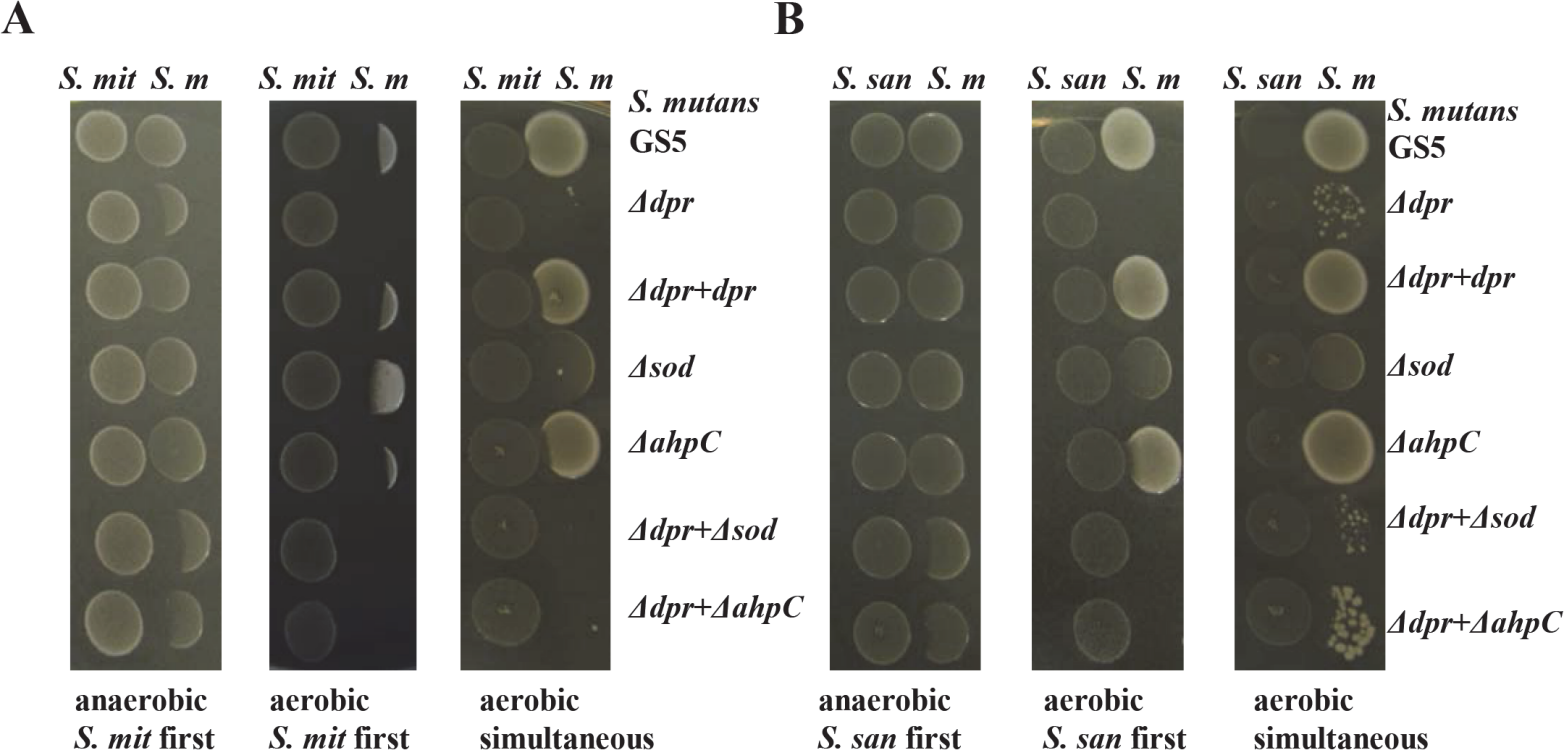
Inhibition of the growth of *S*. *mutans* strains by (A) *S*. *mitis* and (B) *S*. *sanguinis*. *S*. *mitis* or *S*. *sanguinis* was inoculated first and grown for 24 h at 37°C in an anaerobic (anaerobic *S*. *mit*/*S*. *san* first) or aerobic (aerobic *S*. *mit*/*S*. *san* first) atmosphere. Then, *S*. *mutans* strains were inoculated next to these colonizers, and the plates were incubated for 24 h. The *S*. *mitis* or *S*. *sanguinis* strain and *S*. *mutans* were inoculated simultaneously on the plate and incubated for 24 h at 37°C under aerobic conditions (simultaneous aerobic). *S*. *m*, *S*. *mutans*; *S*. *mit*, *S*. *mitis*; *S*. *san*, *S*. *sanguinis*.

In addition, similar experiments were performed using *S*. *mitis* and *S*. *sanguinis*. Under anaerobic conditions, *S*. *mitis* and *S*. *sanguinis* inhibited all of the Δ*dpr* mutants (Δ*dpr*, Δ*dpr* plus Δ*ahpC*, and Δ*dpr* plus Δ*sod*) but not other strains. Under aerobic conditions, when *S*. *mitis* was inoculated first, almost all strains were inhibited (Fig. 3A), while all of the Δ*dpr* and its derivertive mutants were more inhibited compared to the Δ*ahpC* and Δ*sod* mutants (Fig. 3A). When *S*. *sanguinis* was inoculated first, *dpr*-related mutants were more inhibited compared to Δ*ahpC* and Δ*sod* (Fig. 3B). In addition, simultaneous inoculation of *S*. *mutans* strains with initial colonizers led to less growth inhibition in all non-*dpr* mutant *S*. *mutans* strains, and all *dpr*-related mutants were more inhibited (Fig. 3AB). Furthermore, when strains were inoculated adjacent to various concentrations of H_2_O_2_, only *S*. *mutans* Δ*dpr-*defective strains were inhibited (at H_2_O_2_ concentrations of 0.025% to 0.3%) (S4 Fig.).

### Transcriptional analysis of the genes responsible for resistance to oxidative stress

**Fig 4:**
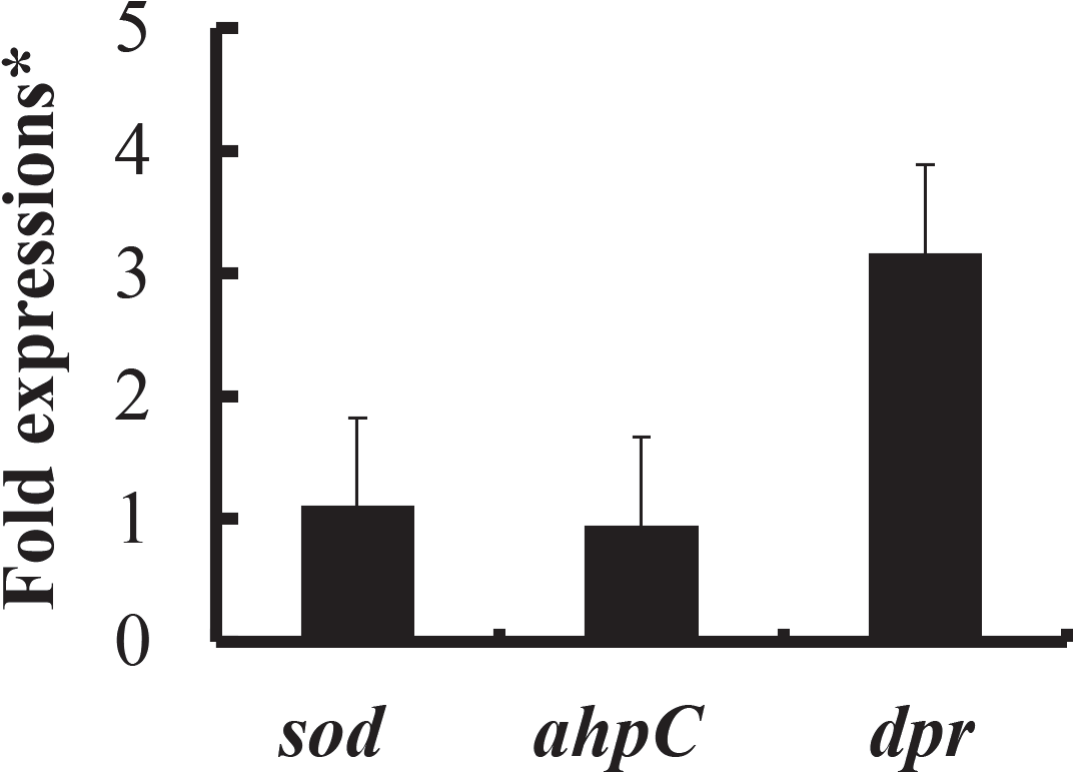
The relative quantities of *S*. *mutans sod*, *ahpC*, and *dpr* genes when co-cultured with *S*. *gordonii*. Fold expressions were shown as the ratio of *S*. *mutans* co-cultured with *S*. *gordonii* to *S*. *mutans* cultured without *S*. *gordonii*. All gene expressions were normalized to *gryA*. The data are expressed as the means and SDs of three experiments.

Of the genes responsible for resistance to oxidative stress, we investigated the expression levels of *ahpC*, *sod*, and *dpr* in *S*. *mutans* with or without *S*. *gordonii*. The expression level of *dpr* in *S*. *mutans* with *S*. *gordonii* was increased 3.2-fold compared to *S*. *mutans* alone, while transcriptional levels of *ahpC* and *sod* in *S*. *mutans* with *S*. *gordonii* were similar to that of *S*. *mutans* without *S*. *gordonii* (Fig. 4).

### Dpr expression in biofilm and planktonic phase

**Fig 5:**
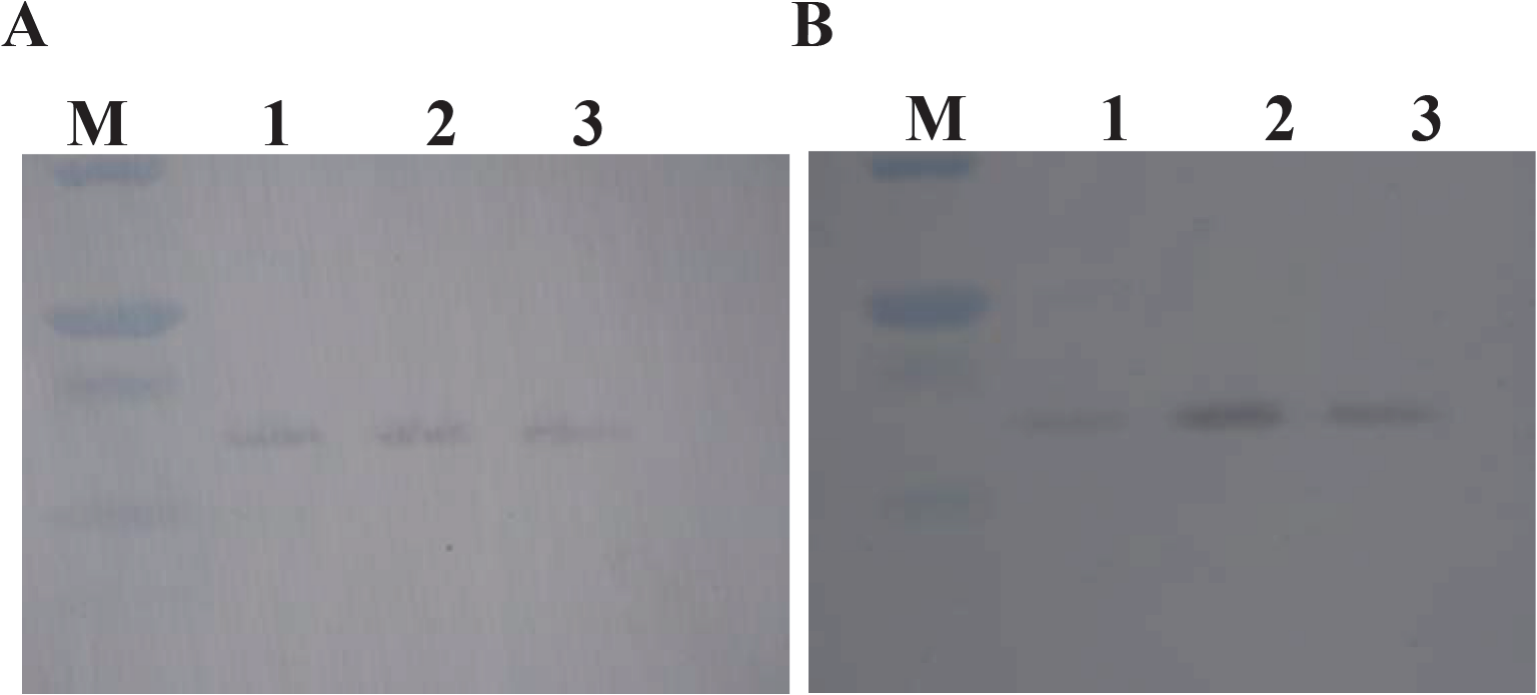
*S*. *mutans* Dpr expression by Western blotting analysis. (A) Planktonic cells. (B) Biofilm. Lane M, molecular mass markers; lane 1, *S*. *mutans* without *S*. *gordonii*; lane 2, *S*. *mutans* co-cultured with *S*. *gordonii* DL1; lane 3, *S*. *mutans* co-cultured with *S*. *gordonii* Δ*spxB*.

Western blot analysis of *S*. *mutans* Dpr expression with/without *S*. *gordonii* was performed. In planktonic cells, all protein expressions were almost same (Fig. 5A), while in *S*. *mutans* biofilm, Dpr expression was most increased when co-inoculated with *S*. *gordonii* compared to inoculated *S*. *mutans* alone and/or with *S*. *gordonii* Δ*spxB* (Fig. 5B).

### Viability assay of *S*. *mutans* strains after co-inoculation with *S*. *gordonii*

**Fig 6:**
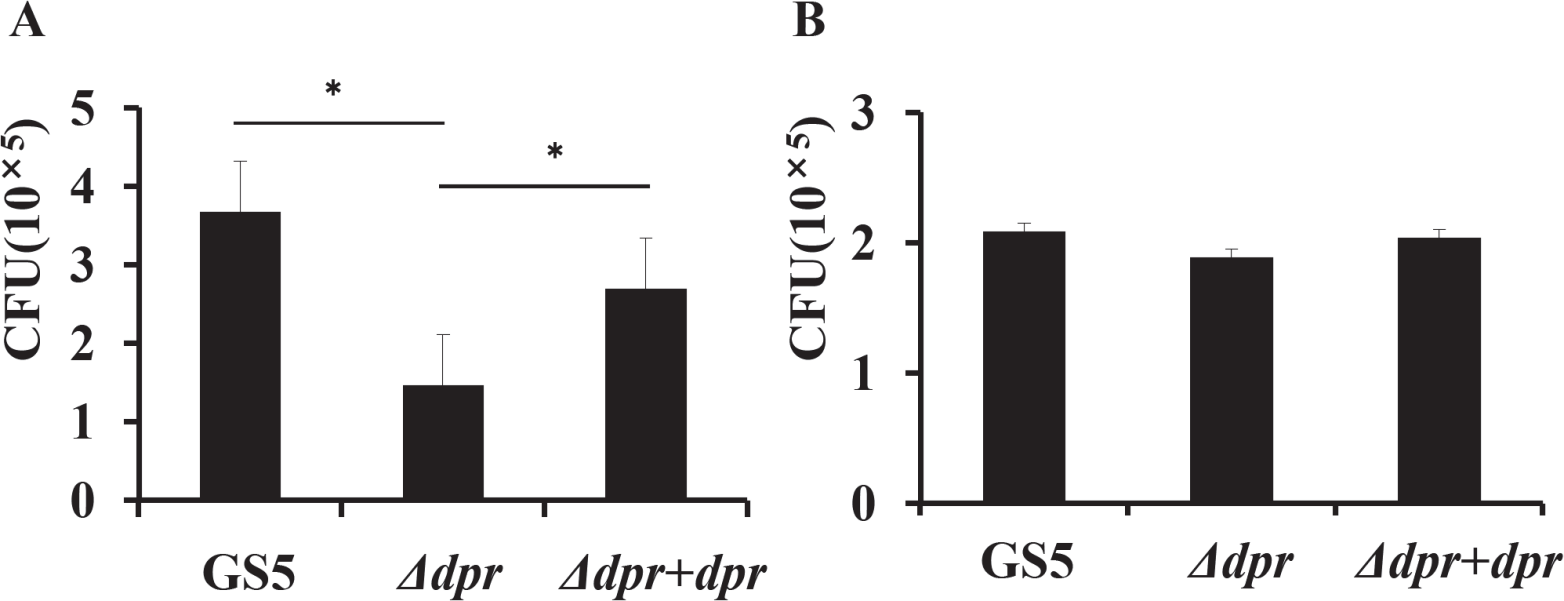
Viability assay of *S*. *mutans* strains after co-inoculation with *S*. *gordonii*. *S*. *mutans* strains were inoculated into *S*. *gordonii* pre-existing BHI medium and additionally inoculated at 37°C under anaerobic conditions for 48 h. *S*. *mutans* CFU on MS agar plates supplemented with bacitracin were counted and adjusted to CFU/well. Data are shown as the means of triplicate platings from one of two reproducible experiments. *P < 0.05.

The viability of the *S*. *mutans* Δ*dpr* mutant with *S*. *gordonii* was attenuated compared to GS5 viability with *S*. *gordonii* (P < 0.05, Fig. 6A), whereas that of the *S*. *mutans dpr*-complemented strain was similar to that of the wild-type GS5 strain (Fig. 6B).

## Discussion

Oral biofilm formation on the tooth surface is considered to be a sequential process involving oral bacteria. Initially, the tooth surface is colonized by a group of bacteria called “pioneer colonizers,” which are mostly composed of mitis group streptococci (*e*.*g*., *S*. *gordonii*, *S*. *sanguinis*, and *S*. *mitis*). Previous investigation reported some *S*. *gordonii* species appears in more mature plques while *S*. *sanginis* and *S*. *oralis* appear in the more initial plaques [4]. Early colonizers such as *S*. *mutans* subsequently adhere to these pioneer colonizers [3, 4]. The growth of these early colonizers modifies the local environment and allows for growth of late colonizers [29]. In this context, several investigations of the interactions between mitis group streptococci and *S*. *mutans* have been reported [29]. In this study, we focused on the interactions between *S*. *mutans* and *S*. *gordonii*. To analyze the proteins upregulated when *S*. *mutans* interacts with *S*. *gordonii*, we performed 2-DE analysis with LC-MS/MS. Proteomic analysis revealed that the most upregulated *S*. *mutans* protein was Dpr, a peroxide resistance protein. In addition, Western blotting analysis revealed that Dpr expression in *S*. *mutans* biofilms was increased when co-cultured with *S*. *gordonii* compared to *S*. *mutans* monoculture (S4 Fig.). Dpr was previously identified as a ferritin-like peroxide resistance protein that incorporates iron ions [30–32]. However, the role of this molecule in the context of the ecological system of oral biofilms has not been reported. In this study, *S*. *mutans* Dpr was the most upregulated protein in biofilms co-cultured with *S*. *gordonii*. In addition, previous studies have reported that some strains and/or species of *S*. *sanguinis* and *S*. *gordonii* antagonize the growth of *S*. *mutans* by the production of H_2_O_2_ [7]. In this regard, upregulation of Dpr in *S*. *mutans* when co-cultured with *S*. *gordonii* is not unexpected.

**Fig 7:**
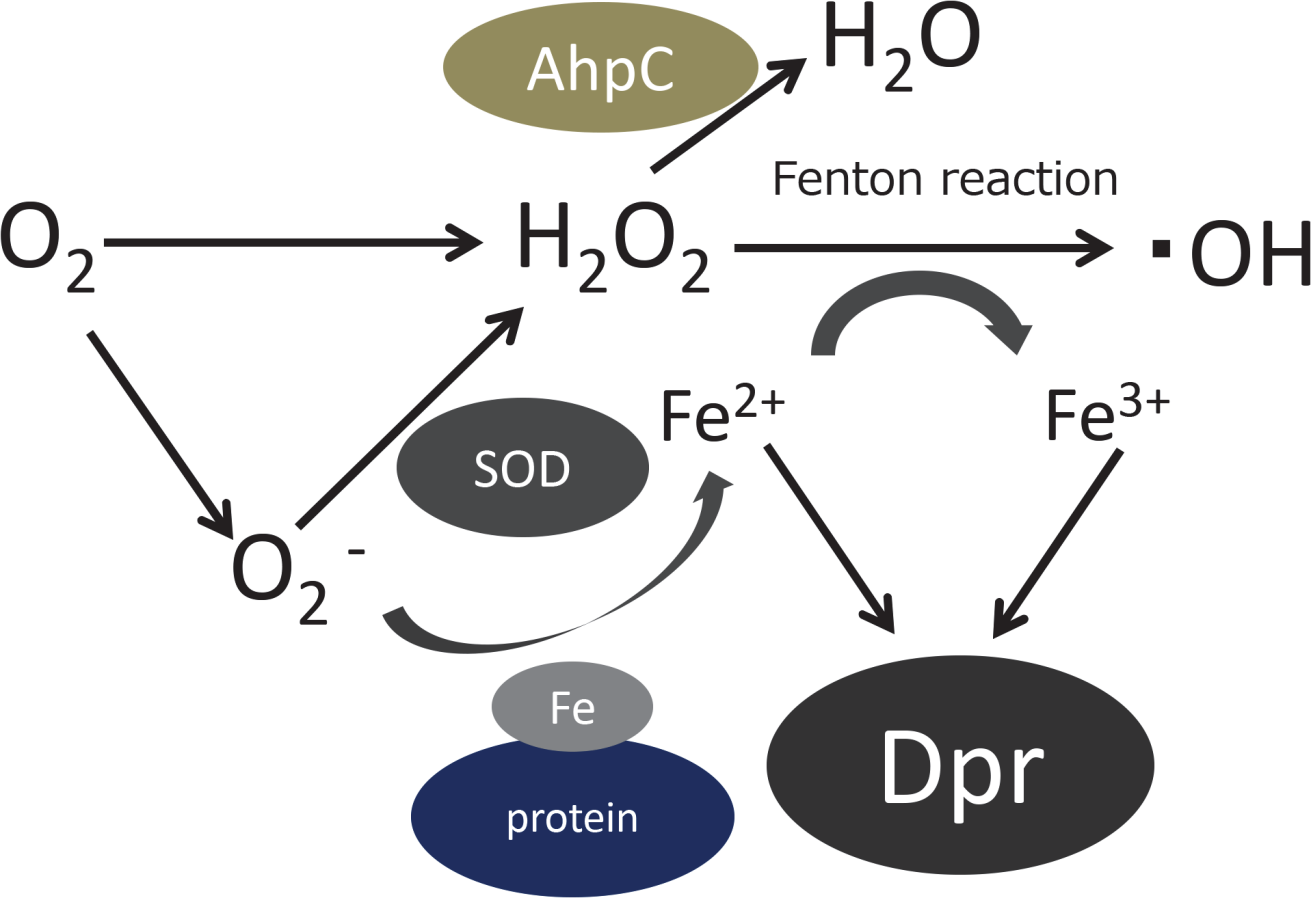
Linkage among iron, Dpr, and oxygen metabolism in *S*. *mutans* [41].

Based on these results, to analyze the role of *S*. *mutans* Dpr protein when encountering pioneer colonizers, we constructed a *dpr*-mutant of *S*. *mutans* UA159 before starting the competition assays. To minimize the effects of growth differences, the growth of all *S*. *mutans* strains was analyzed. Growth of the *dpr*-defective mutant of the *S*. *mutans* UA159 strain was very slow (S1 Fig.). Therefore, we used the *dpr*-defective mutant of *S*. *mutans* GS5 for further analysis; the growth of this strain was almost identical to that of GS5. The competition assay revealed that growth of the Δ*dpr* mutant was more inhibited by initial colonizers compared to the growth of the wild-type GS5 strain. Previous investigations have reported that aerobic conditions increase H_2_O_2_ production of pioneer colonizers [7]. Under aerobic conditions, inhibition of the *S*. *mutans* strains by pioneer colonizers was more enhanced in this study. We additionally analyzed the effects of the mutation in other oxidative stress genes, *ahpC* and *sod*, in *S*. *mutans*. As shown in Fig. 7, H_2_O_2_ produced by Nox-1 (H_2_O_2_ forming NADH oxidase) can be reduced to H_2_O by AhpC [33], while SOD dismutates superoxide (O_2_^-^) to molecular oxygen (O_2_) and H_2_O_2_ [34] (Fig. 7). The mutants in *ahpC* or *sod* gene in *S*. *mutans* were not inhibited by pioneer colonizers, while *dpr* mutant were inhibited. These results showed that the most crucial antioxidant protein of *S*. *mutans* in the protection against initial colonizers was Dpr. Recent investigation reported that the *dpr* and *sod* mutants showed almost no growth against *S*. *sanguinis* [35]. As the result of competition assay using 0.35% H_2_O_2_, the *dpr* mutant showed almost no growth, while the *sod* mutant displayed slight growth compared with that of *dpr* mutant. In addition, in a quantitative assay using Trypticase soy broth containing 0.04% H_2_O_2_, the UA159 strain demonstrated almost 50% survival after 30 min of incubation, while the *dpr* and *sod* mutants showed 0% and 1% survival, respectively. From these results, they concluded that Dpr and SOD are involved in H_2_O_2_ resistance in *S*. *mutans* [35]. Our results showed that *dpr* inactivation resulted in more sensitive than *sod* inactivation. As shown in Fig. 7, SOD mediates the conversion of O_2_^-^ to H_2_O_2_ and O_2_^-^ derived SOD deficiency enhances the Fenton reaction by releasing Fe^2+^ from iron containing proteins [36, 37]. Sutton and Winterbourn described that the rate-determining step of the oxygen metabolism is: H_2_O_2_ + Fe^2+^ →Fe^3+^+OH. +OH^-^ [38]. This investigation supports that our finding, the *dpr* mutant is more sensitive than the *sod* mutant. To elucidate this finding, we analyzed the transcriptional levels of *sod*, *ahpC*, and *dpr* genes of *S*. *mutans* when co-cultured with *S*. *gordonii*. The *dpr* gene was up-regulated for 3.2-fold compared to *S*. *mutans* alone, while *sod* and *ahpC* genes were not up-regulated.

We further analyzed whether Dpr expression is depending on the bacterial phase or not. Western blotting analysis showed the Dpr expression is specific to biofilm status in this investigation (Fig. 5). To elucidate this phenomenon, H_2_O_2_ concentration in both planktonic and biofilm should be monitored. We finally examined the role of *S*. *mutans* Dpr protein for survival when cultured with *S*. *gordonii*. The survival of *dpr*-defective mutant was significantly attenuated compared to parental strain. This result shows Dpr is essential for survival when co-exist with *S*. *gordonii*.

In conclusion, we confirmed that Dpr is involved in protecting *S*. *mutans* from H_2_O_2_ produced by oral streptococci. The survival mechanisms of this organism in the presence of H_2_O_2_ producing bacteria might be important factor for the cariogenic property of this organism.

## Supporting Information

S1 Fig Growth curve of *Streptococcus mutans* and its mutants. The strains UA 159 (open triangles), UA 159 Δ*dpr* (closed squares), GS5 (closed triangles), GS5 Δ*dpr* (open circles), and GS5 Δ*dpr*+*dpr* (*dpr* complement strain, open squares) were inoculated, and the OD_595_ was monitored.
(TIF)

S2 Fig Western blotting analysis for confirmation of Dpr expression in *S*. *mutans* mutants. After washing the overnight cultures, samples were extracted for immunoblotting. M, size marker; 1, *S*. *mutans* GS5; 2, *S*. *mutans* GS5 Δ*dpr*; 3, *S*. *mutans* GS5 Δ*dpr*+*dpr*.
(TIF)

S3 Fig Inhibition of the growth of *S*. *mutans sod*-, *ahpC*-, *dpr*- *ahpC*, and *sod* double mutants by *S*. *gordonii* DL1 *spxB*-deficient mutant. The culture conditions were the same as in Fig. 2.
(TIF)

S4 Fig Competition assay with H_2_O_2_. Various concentrations of H_2_O_2_ were spotted on THB agar plate adjacent to *S*. *mutans* strains. Both H_2_O_2_ and *S*. *mutans* strains were spotted nearby at the same time and incubated for 24 h.
(TIF)
