# Supplementary figures and images for: Proteome Analysis Identifies the Dpr Protein of Streptococcus mutans as an Important Factor in the Presence of Early Streptococcal Colonizers of Tooth Surfaces
Source: PLoS One. 2015 Mar 27;10(3):e0121176. doi: 10.1371/journal.pone.0121176 (PMC4376698; doi:10.1371/journal.pone.0121176)

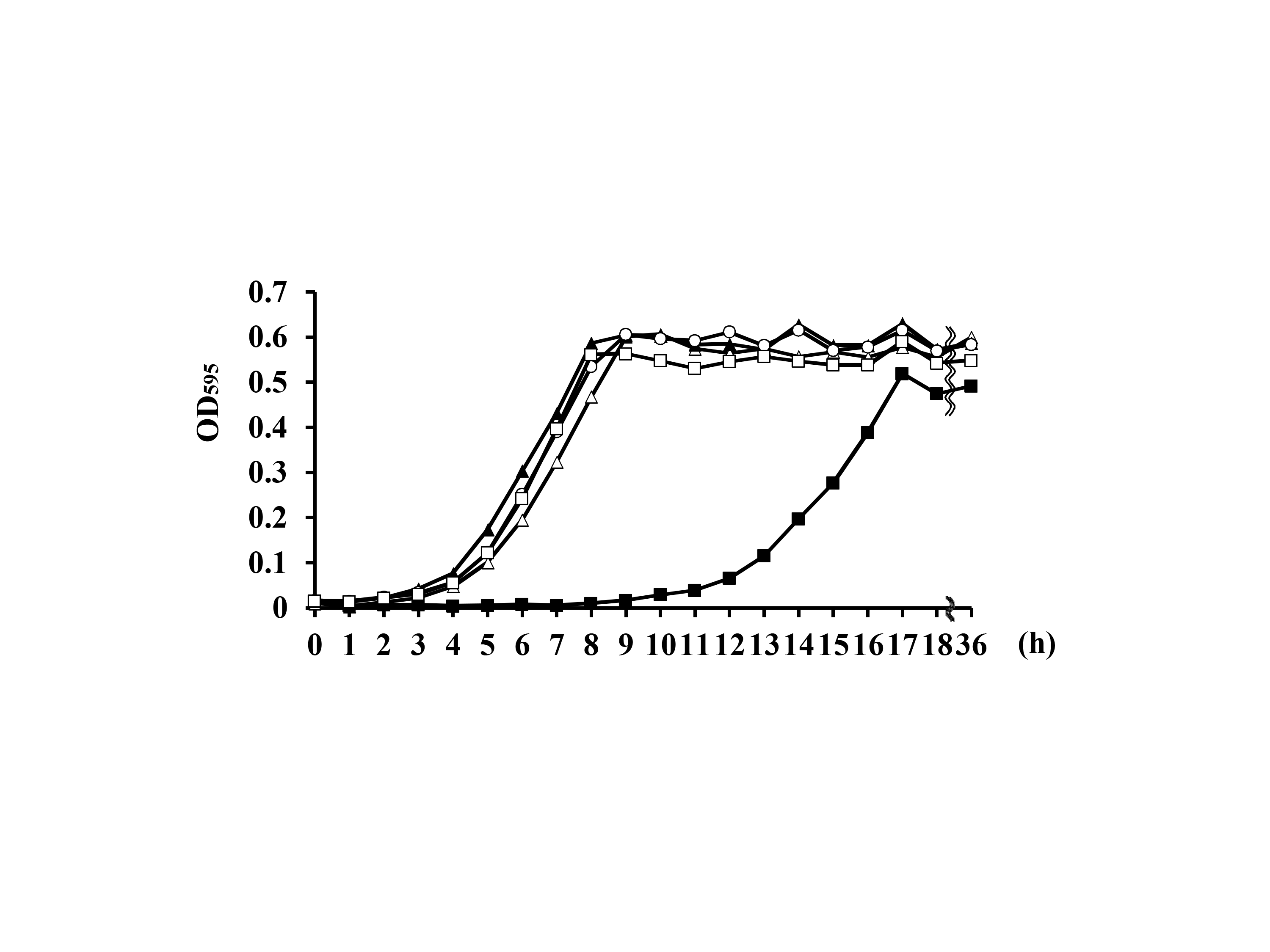

Supplement: S1 Fig — The strains UA 159 (open triangles), UA 159 Δdpr (closed squares), GS5 (closed triangles), GS5 Δdpr (open circles), and GS5 Δdpr+dpr (dpr complement strain, open squares) were inoculated, and the OD595 was monitored. (TIF) [file pone.0121176.s001.tif]

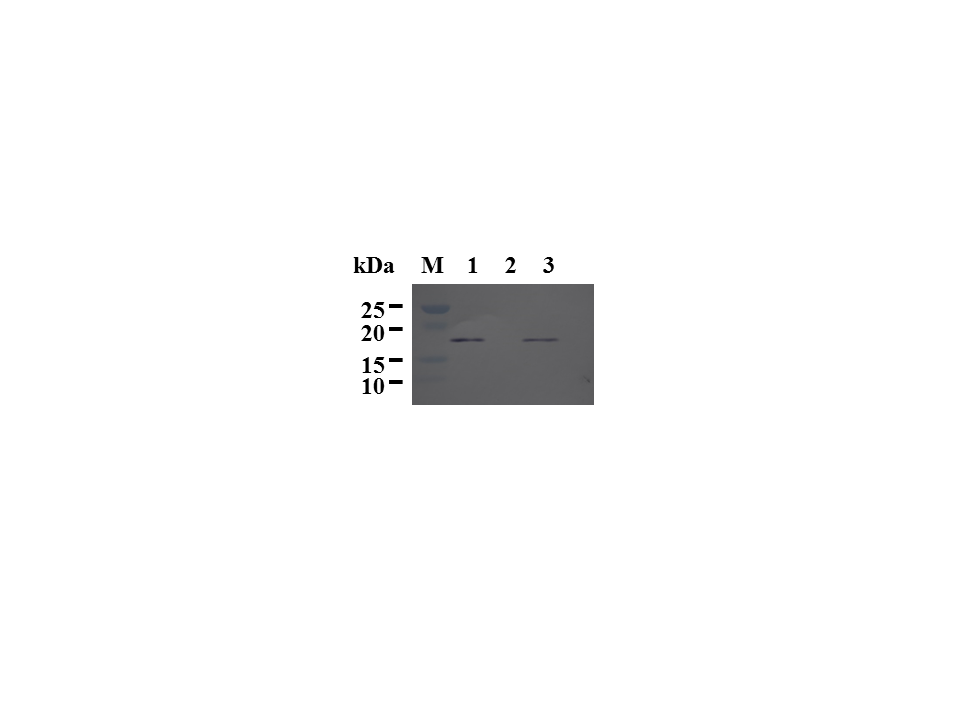

Supplement: S2 Fig — After washing the overnight cultures, samples were extracted for immunoblotting. M, size marker; 1, S. mutans GS5; 2, S. mutans GS5 Δdpr; 3, S. mutans GS5 Δdpr+dpr. (TIF) [file pone.0121176.s002.tif]

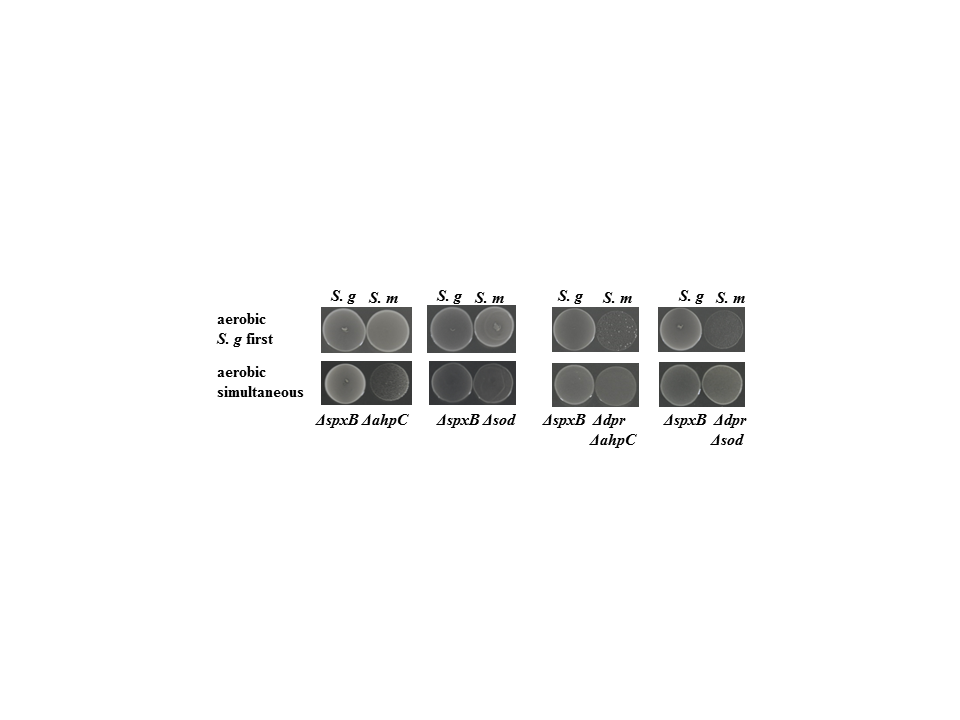

Supplement: S3 Fig — The culture conditions were the same as in Fig. 2. (TIF) [file pone.0121176.s003.tif]

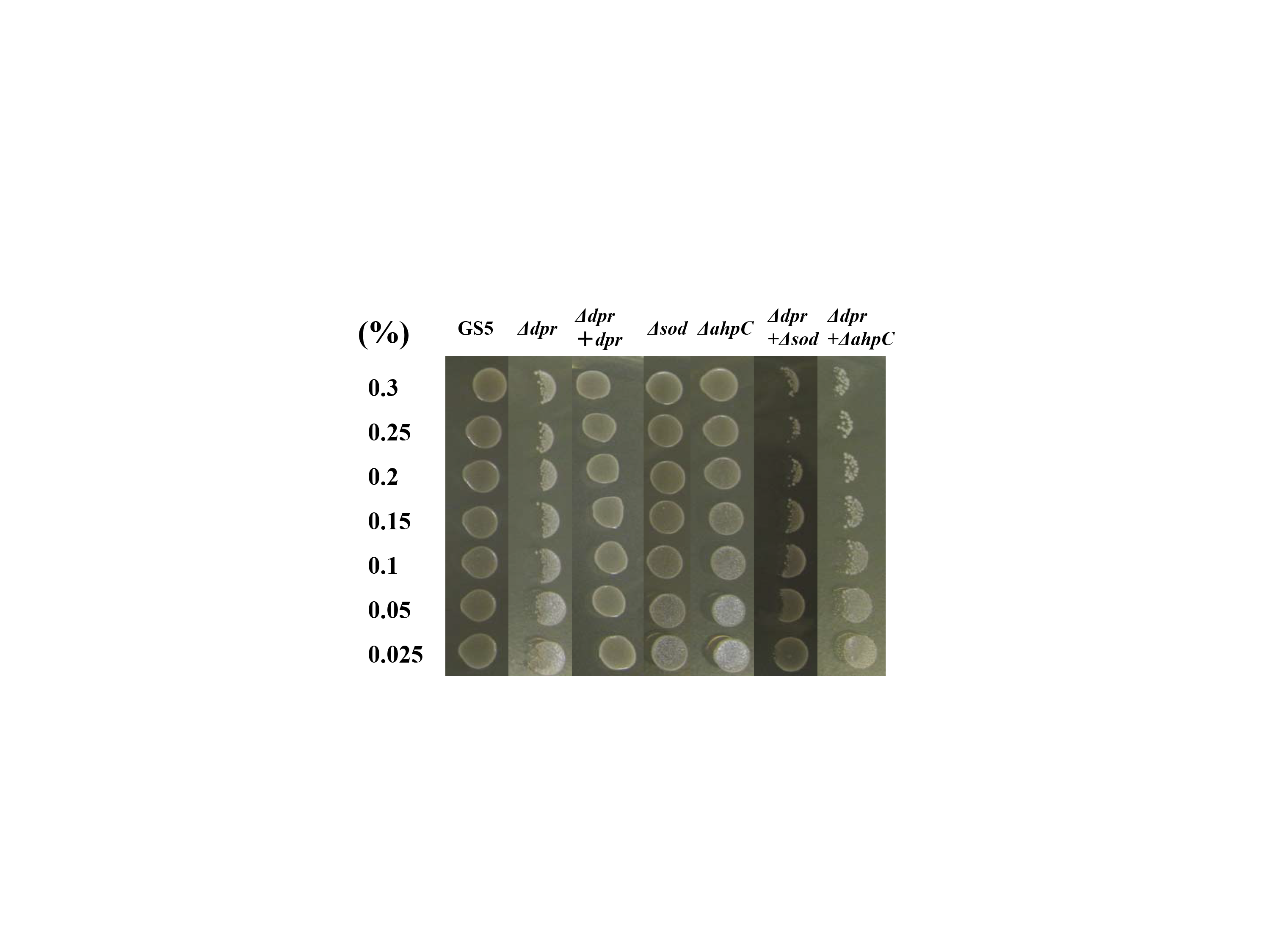

Supplement: S4 Fig — Various concentrations of H2O2 were spotted on THB agar plate adjacent to S. mutans strains. Both H2O2 and S. mutans strains were spotted nearby at the same time and incubated for 24 h. (TIF) [file pone.0121176.s004.tif]
